# Model-Based and Model-Free Social Cognition: Investigating the Role of Habit in Social Attitude Formation and Choice

**DOI:** 10.3389/fpsyg.2019.02592

**Published:** 2019-11-21

**Authors:** Leor M. Hackel, Jeffrey J. Berg, Björn R. Lindström, David M. Amodio

**Affiliations:** ^1^Department of Psychology, University of Southern California, Los Angeles, CA, United States; ^2^Department of Psychology, New York University, New York, NY, United States; ^3^Department of Psychology, University of Amsterdam, Amsterdam, Netherlands

**Keywords:** social, cognition, attitude, learning, habit, model-free, model-based, computational

## Abstract

Do habits play a role in our social impressions? To investigate the contribution of habits to the formation of social attitudes, we examined the roles of model-free and model-based reinforcement learning in social interactions – computations linked in past work to habit and planning, respectively. Participants in this study learned about novel individuals in a sequential reinforcement learning paradigm, choosing financial advisors who led them to high- or low-paying stocks. Results indicated that participants relied on both model-based and model-free learning, such that each type of learning was expressed in both advisor choices and post-task self-reported liking of advisors. Specifically, participants preferred advisors who could provide large future rewards as well as advisors who had provided them with large rewards in the past. Although participants relied more heavily on model-based learning overall, they varied in their use of model-based and model-free learning strategies, and this individual difference influenced the way in which learning related to self-reported attitudes: among participants who relied more on model-free learning, model-free social learning related more to post-task attitudes. We discuss implications for attitudes, trait impressions, and social behavior, as well as the role of habits in a memory systems model of social cognition.

## Model-Based and Model-Free Social Cognition

Human thriving depends on social relationships, and the impressions we form of new acquaintances are essential guides to our social behavior (Fitzsimons and Anderson, 2013). We befriend people who are kind, hire people who are competent, avoid those who are domineering, or seek counsel from those who are empathic. In this way, impression formation often serves our goals (Brewer, 1988; Fiske and Neuberg, 1990; Bargh and Ferguson, 2000), as we use our knowledge of other people – their traits, mental states, and behaviors – to predict their actions and decide whether to interact with them (Heider, 1958; Tamir and Thornton, 2018).

Yet, while goals drive much of human behavior, this is not always the case. Habits, in particular, are responses that occur automatically and independent of our goals, often representing a highly-repeated behavior that was once goal-directed but that persists and is expressed even when the goal has changed (Wood and Rünger, 2016; Robbins and Costa, 2017). Habits likely explain many behaviors, from benign compulsions like biting one’s nails to more harmful acts like mindlessly reaching for a cigarette. Here, we asked whether habit-like processes may also contribute to social cognition – how we learn about, interact with, and evaluate other people – and thus help explain social behaviors that appear to occur independently of, or in opposition to, one’s goals.

### Multiple Systems for Social Learning

Research on impression formation has, to date, primarily emphasized conceptual forms of learning that give rise to goal-directed behavior; that is, acquiring conceptual knowledge about a person’s traits and behavior (Uleman and Kressel, 2013). Early theories of impression formation focused on instructed forms of learning, in which we learn about a person from descriptions shared by others (Asch, 1946; Wyer and Carlston, 1979). If we are told that Bob is generous and friendly, we may infer that he’s a good person. We can also learn about other people through observation and the use of attributional processing (Heider, 1958; Jones and Davis, 1965; Rydell and McConnell, 2006). If we see Jane offer money to a homeless person, we may infer from her actions that she is generous; if we see Jane choose a high-performing stock, we may infer that she is competent. These conceptual inferences can give rise to goal-directed behaviors, like choosing to spend time with someone who is generous or to hire someone who is competent.

More recent research has shown that social attitudes and impressions can also be formed through reward-based instrumental learning in direct social interaction – trial-and-error learning in which people make choices and receive feedback (Hackel et al., 2015). For instance, one might choose a lunch partner and experience rewards when they share their food, or one might hire a financial advisor and experience rewards when their advice pays off. Through this feedback, one can learn the reward value of an individual while also inferring aspects of their character traits (Hackel et al., 2015). Unlike instructed and observational forms of learning, which are typically passive (e.g., reading about another person), instrumental learning is active: it concerns feedback from another person regarding one’s own actions. If, on most days, Bob’s greeting to Jane is met with a smile, he will associate reward with his behavior toward Jane in addition to inferring that she is friendly.

Instrumental learning thus represents a distinct mode of learning in social interactions relative to conceptual knowledge (Amodio, 2019). Instead of inferring other people’s qualities in order to decide how to interact with them, instrumental learning involves learning the reward value of social interaction through direct action and feedback. That is, in traditional impression formation approaches, Bob learns to interact with Jane because he infers she is friendly, and he wants to be around friendly people. In instrumental learning, Bob learns to interact with Jane because he previously did so and received rewarding outcomes, such as social rewards like smiles and compliments or material rewards like money and food. He may like Jane as a result of those rewards, rather than as a result of qualities he attributes to her. Thus, instrumental learning directly informs how we should interact with others given the rewards they provide. In this way, preferences acquired through instrumental learning may be more directly tied to behavior.

### A Role for Habits in Social Cognition?

Over time, instrumentally learned responses may be automatized into habits (Thorndike, 1911; Robbins and Costa, 2017). Although people may initially perform an action deliberately to achieve a goal, rewards can “stamp in” an association between a stimulus (or context) and a response, such that people later perform the response automatically. In contrast to skills, which are goal-directed action routines triggered intentionally, habits reflect a well-learned response that unfolds even when it is not consistent with a goal, and it persists even when its expression is no longer rewarded (Balleine and Dickinson, 1998; Tricomi et al., 2009; Wood and Rünger, 2016; Wood, 2017). Nevertheless, habits can be adaptive, initiating an important behavior that we might otherwise forget in the pursuit of another goal, such as grabbing our keys when rushing out the door to get to work in the morning.

Habits differ from other forms of unintentional learning that may contribute to impression formation. For example, spontaneous trait impressions (STIs) form when a perceiver is simply asked to read and memorize a set of trait-implying sentences (Winter and Uleman, 1984; Carlston and Skowronski, 1994). People may be unaware that they formed an impression, yet STIs become evident in measures of cued recall and may subsequently influence judgment (Moskowitz and Roman, 1992). There is also evidence that evaluative conditioning, in which a neutral social target is paired repeatedly with either positive or negative images (Walther, 2002; Olson and Fazio, 2006), may even occur when such images are presented subliminally (e.g., De Houwer et al., 1997; Hofmann et al., 2010; but see Sweldens et al., 2014). However, both forms of learning involve passive exposure to stimuli and the formation of conceptual associations, likely supported by a semantic/conceptual associative memory system (Amodio and Berg, 2018; Amodio, 2019), in contrast to the active process of action-outcome learning involved in instrumental habit formation.

### Examining Habit Formation Through Reinforcement Learning

A major challenge in the study of habits in humans is that it is often difficult to discern habits from other, goal-directed processes in behavior. However, this distinction has recently been linked to two forms of behavior within a computational account of reinforcement learning (Daw et al., 2011). Broadly, reinforcement learning algorithms describe how an agent learns the value of different actions with different states of the world by making choices and experiencing rewards (Sutton and Barto, 1998). According to this account, two types of computations can underlie reinforcement learning: Agents can engage in *model-based learning*, in which they consider the likely outcomes of their actions given knowledge about their environment, and also in *model-free* learning, in which they associate actions directly with reward value and repeat previously rewarded actions (Daw et al., 2011; Doll et al., 2015). Model-based learning is thus prospective and goal-oriented, sensitive to both environmental contingencies (e.g., how to get to a reward) and expected outcomes (e.g., whether a desirable reward will be attained) – like a hungry mouse considering how to navigate a maze to reach the room with the tastiest cheese. In contrast, model-free learning is retrospective, relying on a past history of rewards for an action; it requires no internal model of one’s environment and is insensitive to the outcomes an action will presently bring. A model-free learner stores cached values for previously performed actions and selects actions with the highest cached value.

Because model-free learning is computationally simpler but less flexible than model-based learning, it may give rise to behavior that has features of habits. For instance, an animal might continue to press a food lever despite being fully sated because this action was previously rewarded and thus associated with high reward value (Dickinson and Balleine, 1994; Daw et al., 2011). Although a model-free learner could eventually learn to adapt to the new value, it would persist in pressing the lever until learning takes place in its newly satiated state. In contrast, a model-based learner should not require this learning at all; instead, it should plan ahead to the likely outcome of the lever press, realize that it does not desire that outcome, and avoid the action from the start. Given these characteristics, the model-based/model-free distinction has been used recently to probe the role of habits in a range of learning contexts in humans. For instance, individuals who engage in greater model-based learning show less persistence in a devaluation task – a classic marker of habits (Gillan et al., 2015). Yet, to date, this approach has not been applied to questions on the formation of social impressions through direct social interactions with other people.

### Model-Free Learning in Social Cognition

How might a model-based/model-free account relate to social impressions? When other people provide us with material feedback (like a gift) or social feedback (like a smile or a compliment), we experience this feedback as rewarding; as a result, this feedback can reinforce our social choices and draw us back to the same partners again in the future (Jones et al., 2011; Lin et al., 2011; Lindström et al., 2014; Hackel et al., 2015; Lindström and Tobler, 2018). If people learn from this feedback in a model-free manner, specifically, they might return to interaction partners previously associated with high reward regardless of whether those partners will currently provide desirable outcomes. This pattern would resemble a traditional definition of habit.

Some existing work hints at the possibility that reward feedback gives rise to social preferences that persist in a habit-like manner. In research by Hackel et al. (2015), participants played an economic game in which they chose partners who could share money; partners varied in the average *amount* they shared (indicating reward value) and average *proportion* they shared (indicating generosity). During initial learning, it was economically advantageous for participants to prefer individuals who provided large rewards, regardless of their generosity. However, when participants were later asked to choose one of these partners to work with in a non-economic puzzle-solving task – a context where generosity, but not previous reward value, is advantageous – participants’ choices were still influenced by partners’ past reward value in addition to their generosity. This persistent influence of past reward – even when reward value no longer informed desired outcomes – suggests that participants may have developed model-free reward associations that guided subsequent social preferences. Nevertheless, past work has not directly tested this possibility by dissociating model-based and model-free learning in social interaction.

### Study Overview

The present research was designed to provide initial evidence for model-free learning in social impression formation. To this end, we administered a sequential choice task commonly used to dissociate model-based and model-free learning (Kool et al., 2016; Kool et al., 2017; see also Daw et al., 2011), adapted to examine social partner choice and attitudes. On each round, participants chose financial advisors who had supposedly invested in one of two stocks; participants then received a payout from that advisor’s stock. We examined the extent to which participants chose advisors based on model-based and model-free reinforcement, and further examined whether these forms of learning predicted participants’ subjective attitude toward each advisor.

## Materials and Methods

### Participants

Sixty-nine participants (42 male, 27 female) were recruited via Amazon Mechanical Turk (AMT), in exchange for $3.50 for study completion, plus a monetary bonus based on their task performance. A sample size of 65 participants was chosen *a priori*; an additional four participants completed the task due to an error in which an extra set of slots was posted. Data collection was completed before analysis. Participants were eligible if they were located in the United States, completed at least one prior AMT study, and had approval rates of at least 95%. Informed consent was obtained from all participants in accordance with the guidelines of the New York University Committee on Activities Involving Human Subjects. We excluded data from participants who did not respond in time to either the first or second stage of a trial on more than 20% of trials (Kool et al., 2017). This rule excluded data from four participants, leaving data from 65 participants in analyses.

### Procedure

Participation took place via Psiturk, an online platform for cognitive tasks (Gureckis et al., 2016). After providing consent, participants read a self-guided description of the study, which included practice trials, and completed the main experimental task. Next, participants completed self-reported evaluation items and a demographics questionnaire. Lastly, participants were informed of their bonus compensation for participating and then completed a debriefing procedure that included a suspicion probe and an explanation of study goals. All data exclusions, all manipulations, and all measures included in this research are fully reported in this article.

#### Two-Step Task

**FIGURE 1:**
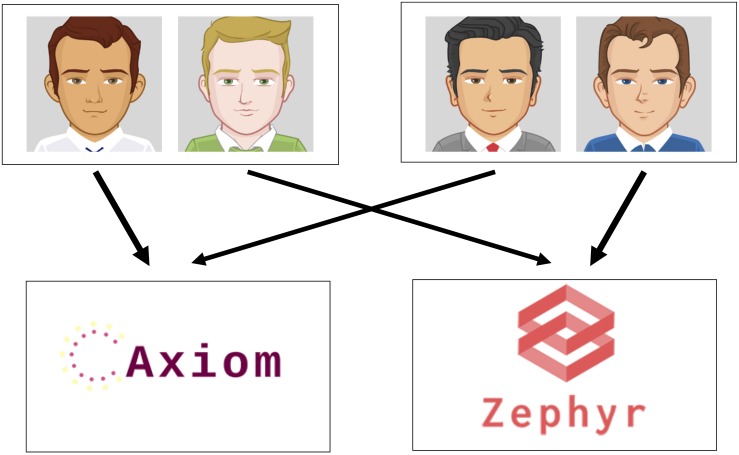
Schematic of task design. In the first stage of each round, participants saw one of two sets of advisors and chose an advisor for that round. Participants then viewed the stock that advisor had chosen; after making a button press, participants saw feedback indicating the payout provided by the stock, ranging from zero to nine points. Within each pair of advisors, one advisor always led to the “Axiom” stock and the other always led to the “Zephyr” stock. This feature of the task rendered the two sets of advisors equivalent, such that a model-based learner could apply experiences with one set of advisors to choices involving the other set of advisors.

We adapted a sequential learning task (Kool et al., 2016, 2017) designed to dissociate model-free and model-based learning (Figure 1). In our adaptation, participants were told they would learn about choices made by four AMT workers who previously participated in a financial decision-making study (see Supplementary Material for full task instructions). According to this cover story, these previous workers were assigned the role of “Financial Advisor,” in which they chose (only) one of two stocks (“Axiom” and “Zephyr”) to invest in for the duration of the study. These Advisors then earned money based on the performance of their chosen stock, which fluctuated throughout the study and could change from one round of “dividends” to the next.

Next, participants were assigned to the role of the “Client,” in which they would make a series of decisions about which Advisor to hire. Participants learned they would earn points based on the performance of the stock chosen by their hired Advisor on each round. Participants were explicitly told that the performance of the stocks would change over time (“a stock that was bad at the beginning of the game might start performing well, and a stock that initially pays well might perform poorly later on”), and that they should try to hire Advisors with the better performing stock at that particular moment. Moreover, participants were informed that they would receive a monetary bonus for their performance in the task, with better performance (in terms of points earned) equating to a larger bonus.

Return on each trial, participants began in one of two randomly chosen first-stage states. In these states, participants were presented with one of two pairs of Advisors, represented by distinct cartoon avatars (Figure 1). Avatars were randomly assigned to different roles across participants (i.e., which stock they were linked with) and were equally likely to appear on the left or right side of the screen. Participants chose one of the two Advisors via button response and then transitioned deterministically to one of the two stocks, which comprised the second-stage states. That is, participants could reach either of the two stocks from each of the first-stage states; one Advisor in each pair always invested in the Axiom stock and the other Advisor in the given pair always invested in the Zephyr stock.

When they reached the second-stage state, participants were instructed to press the spacebar to reveal the performance of the stock in which the chosen Advisor invested. If participants did not respond in time to either the first- or second-stage states, no reward was provided and participants moved to the next trial. The number of points obtained for each stock fluctuated slowly and stochastically over the course of the task, varying according to a Gaussian random walk (*SD* = 2) with reflecting bounds at 0 and +9 points. The drifting nature of the reward feedback encouraged continuous learning throughout the task.

Importantly, the two first-stage states were equivalent in terms of the stocks they could lead to: within each pair of advisors, one Advisor always invested in the Axiom stock, whereas the other Advisor always invested in the Zephyr stock. This design allows for the separation of model-free and model-based control. Given that both stocks can be reached from each pair of Advisors, the stock reached from one set of advisors can be used by a model-based learner to update preferences regardless of which set of advisors is encountered on the next trial. For instance, if an Advisor in one pair invested in the Axiom stock and this stock paid out a large number of points on that trial, a model-based learner should subsequently be more likely to choose the Advisor in the other pair that also invests in the Axiom stock. That is, a model-based learner can generalize across equivalent first-stage choice options due to its exploitation of the overarching task structure. Conversely, model-free learners would not generalize across equivalent first-stage choice options, as they simply rely on directly-experienced action-outcome associations – the outcomes experienced following a choice in one pair of advisors should not affect preferences for the advisors in the second pair, and vice-versa.

Participants were trained extensively on the deterministic transitions (i.e., which financial advisor in a given pairing invested in which of the two stocks) prior to completing the experimental trials, such that 80% accuracy across 15 consecutive trials was required to advance to the main task. Participants did not receive explicit instructions on which advisor led to which stock, but rather were required to learn these transitions through experience. After this training phase, participants completed 150 trials of the main task, split evenly between the two first-stage states. The response deadline in both stages was 1500 ms and feedback was presented for 1000 ms.

#### Post-task Evaluations

Following the two-step task, participants responded to a series of self-report items which pertained to participants’ evaluations (or “liking”) of the different Advisors encountered during the two-step task. Participants were presented with the avatar of each financial advisor, one at a time, and rated how much they liked the advisor using a seven-point scale (from 1 = “Do not like them at all” to 7 = “Like them a lot”). Finally, participants were also asked to estimate how valuable, on average, each of the two stocks were over the course of the learning task (see Supplementary Material).

### Computational Model

In order to determine the degree to which participants employed model-based and model-free learning, we fit data from the learning phase to a computational model of reinforcement learning used in previous work (Kool et al., 2017). Doing so allowed us to estimate latent variables related to social learning for each subject (Hackel and Amodio, 2018), which we then used as input in our analyses.

The model contains a hybrid of model-free learning and model-based learning for selecting advisors (see Supplementary Material for additional details and Supplementary Table S1 for parameter fits). The model-free system stores values for advisors at the first stage and for stocks at the second stage based on prior reward feedback. The model-based system computes the value of selecting each advisor at the time of choice, combining knowledge about how advisors lead to stocks with the expected payoff of each stock (acquired through model-free learning at the second stage). A model-based learner thus prospectively plans toward a goal: he or she selects an advisor based on the stock the advisor will lead to, in light of the reward expected from each stock. In contrast, a model-free learner selects advisors based on the rewards those advisors have led to in the past.

Critically, the model includes a weighting parameter (*w*) that indicates the relative influence of model-based and model-free learning in choice, ranging between 0 (purely model-free) and 1 (purely model-based). This parameter can serve as an individual difference measure of the extent to which a participant engaged in model-based or model-free learning. We fit this model for each participant using maximum *a posteriori* (MAP) estimation, with empirical priors used in previous work (Gershman, 2016; Kool et al., 2017). Doing so allowed us to estimate each participant’s *w* parameter (mean = 0.83), indicating the extent to which they relied on model-based vs. model-free learning. We used this parameter in subsequent analyses examining individual differences in the use of these learning strategies.

## Results

### Model-Free and Model-Based Social Learning

To what extent did participants engage in model-based and model-free social learning? To answer this question, we examined choices in the learning phase, drawing on the following logic of the task. As noted above, the two sets of advisors in the task are equivalent, such that one advisor from each set leads to a particular stock. As a result, a model-based learner would generalize experiences with one set of advisors to the other set. For instance, imagine a participant who sees the first pair of advisors, picks the advisor that leads to the “Axiom” stock, and receives a large reward. On the next round, a model-based learner would try to return to the “Axiom” stock regardless of whether they see the same pair of advisors or a different pair of advisors. In contrast, a model-free learner updates values for individual advisors and chooses advisors based on these values. A model-free learner would therefore repeat their choice on the next trial if presented with the same advisors but would do so to a lesser extent if presented with different advisors. That is, the model-free learner would fail to generalize across sets of advisors.

**FIGURE 2:**
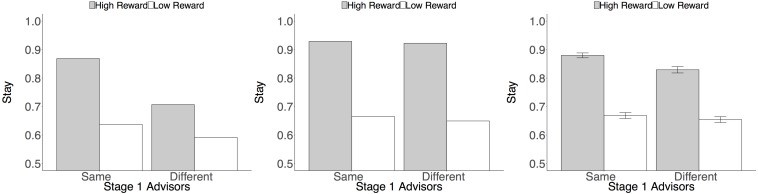
Behavioral predictions and data. Plots depict the probability of staying with the same second-stage stock as on the previous trial, based on whether the set of advisors encountered at the first stage was the same as or different from that of the previous trial, and whether the previous trial delivered a high or low reward. **(A)** Simulated model-based predictions. **(B)** Simulated model-free predictions. **(A,B)** Produced from model simulations (see section Supplementary Material) with weighting parameter *w* specifying fully model-based (*w* = 1) and fully model-free learning (*w* = 0), respectively. **(C)** Observed data indicates the presence of both model-based and model-free learning. Error bars reflect standard error of the mean, adjusted for within-subjects comparisons (Morey, 2008).

Drawing on this task logic, we fit learning phase data to a lagged regression model predicting, on a trial-by-trial basis, whether or not participants repeated their most recent choice of Stage 2 stocks (1 = stay, 0 = switch). This analysis provides a model-agnostic way to test the qualitative behavioral predictions of the model-free/model-based account of learning. Following Kool et al. (2016), predictors included the reward earned on the previous trial (standardized, within-subject, to z-scores), whether or not the previous trial started with the same set of advisors (1 = same, -1 = different), and the interaction of these two predictors. A main effect of reward would indicate model-based learning: people return to a high-paying stock regardless of whether they see the same or different advisors on the next trial to get to that stock (simulated data shown in Figure 2A). An interaction of reward and start state would indicate model-free learning: people try to return to a high-paying stock, but particularly do so when presented with the same set of advisors, thus repeating the advisor choice that led to the large reward (Figure 2B). Models were fit using the lme4 package in R (Bates et al., 2015; R Core Team, 2016). Random variances were allowed for the intercept and all slopes (see Supplementary Table S2 for all coefficients.).

This analysis revealed a main effect of reward, *b* = 1.47, *SE* = 0.07, *z* = 19.80, *p* < 0.001, consistent with model-based learning: overall, participants returned to second-stage stocks after receiving large rewards. However, the analysis also revealed a Reward × Start State interaction, *b* = 0.22, *SE* = 0.03, *z* = 6.45, *p* < 0.001, indicating the presence of model-free learning: participants were more likely to return to a high-paying stock when starting with the same advisors at the first stage. Although participants in our sample were highly model-based (mean w parameter in the computational model fits = 0.83), these results support the hypothesis that both model-based and model-free reinforcement learning contributed to social choice (Figure 2C).

### Post-task Evaluations

If reinforcement learning also gives rise to attitudes, participants might like advisors who can provide reward in the future (model-based value) and advisors associated with past reward (model-free value). To test how learning affects attitudes, we examined participants’ self-reported liking of each advisor following the learning task. Using each subject’s individual parameter fits in the computational model, we estimated the final model-based and model-free values associated with each advisor for each subject at the end of learning, given the unique series of stimuli and outcomes viewed by each participant. We then regressed liking ratings simultaneously on each type of value.

Notably, model-based values were identical for advisors who led to the same stock. That is, if the Axiom stock would be expected to deliver 6 points on average at the end of the task, then each advisor who leads to the Axiom stock would have a model-based value of 6 points. If social evaluations reflect model-based learning, participants would therefore like the two advisors who led to the Axiom stock equally. In contrast, model-free values reflect the unique reward history associated with a particular advisor; even for two advisors who led to the Axiom stock, participants might have experienced different reward outcomes with each advisor. If social evaluations reflect model-free learning, people would therefore prefer advisors who provided greater rewards. Finally, this tendency should depend on individual differences in learning, as reflected in the *w* parameter: individuals who engage in greater model-free learning should especially like advisors associated with high model-free value.

To test these hypotheses, we fit a mixed-effects linear regression predicting post-task liking ratings (Supplementary Table S3). Predictors included each participant’s final model-free values and model-based values toward each advisor (estimated from the computational model), each participant’s *w* parameter, and the interaction of *w* with each type of value. Each predictor was standardized to z-scores (within-subject for the value regressors and between-subject for the *w* parameter). As a result, main effects of value regressors are interpretable relative to the mean level of the *w* parameter (*w* = 0.83). We included random variances for the intercept and each predictor. The models were fit using the lme4 package and lmerTest packages (Bates et al., 2015; Kuznetsova et al., 2016) in R (R Core Team, 2016).

This analysis yielded a main effect of model-based values, *b* = 0.30, *SE* = 0.14, *t*(71.46) = 2.17, *p* = 0.03, and a marginally significant main effect of model-free values, *b* = 0.16, *SE* = 0.09, *t*(162.97) = 1.82, *p* = 0.07. In other words, at mean levels of the *w* parameter, attitudes reflected both kinds of learning: people liked advisors who could lead them to more rewarding stocks and also liked advisors who were uniquely associated with greater reward in the past.

We further examined whether the effects of model-based and model-free learning on reported attitudes varied by participants’ individual learning tendencies, as indexed by the *w* parameter. We found that the *w* parameter, which represents this individual difference variable, interacted with model-free values, *b* = −0.24, *SE* = 0.08, *t*(148.01) = −2.97, *p* = 0.004. Participants who exhibited relatively greater model-free learning also expressed greater liking of partners who had provided more reward. Simple effects analysis supported this interpretation: for learners relying relatively more on model-free control (centered at the 25th percentile of the *w* parameter, or *w* = 0.70), model-free values were strongly predictive of attitudes toward advisors, *b* = 0.31, *SE* = 0.10, *t*(155.32) = 3.11, *p* = 0.002, revealing a novel effect of model-free learning on social evaluation. By contrast, for those relying relative more on model-based control (centered at the 75th percentile of the *w* parameter, or *w* = 1), model-free values were not associated with evaluations, *b* = −0.03, *SE* = 0.11, *t*(162.01) = −0.31, *p* = 0.76. Thus, participants who exhibited model-free learning also liked advisors associated with greater model-free value^1^.

Together, these results identify two ways in which reinforcement learning influences social attitudes, one that is goal-directed and one that is habit-like: people like others who are equivalently capable of providing large rewards in the future, and they also like others who have uniquely provided large rewards in the past. Moreover, the influence of past (model-free) reward history depends on individual differences in learning: individuals who weight model-free rewards more strongly during learning also have a stronger preference for advisors associated with past rewards.

## Discussion

Does habit play role a social impressions? Our findings demonstrate that, indeed, people form impressions through reward-based reinforcement processes that include model-free learning – a form of learning thought to contribute to habitual behavior. In the sequential learning task used here, participants chose financial advisors based on both model-based and model-free learning. That is, participants chose advisors who could lead them to desirable stocks in the future (model-based) as well as who were associated with high rewards in prior interactions (model-free). Although participants relied far more heavily on model-based (as opposed to model-free learning) in general, this pattern of model-free learning suggests the additional role of a habit-like component of learning and behavior in the context of social impression formation.

Furthermore, participants’ learning processes had implications for their explicit social evaluations. Across participants, both model-based and model-free learning predicted self-reported attitudes toward advisors. Moreover, participants varied in their reliance on model-based vs. model-free processing during the learning task, and this individual difference in learning related to differences in evaluation: participants who exhibited greater model-free learning during the investment task showed an effect of model-free learning on self-reported attitudes. Thus, these findings dissociate two routes through which reinforcement learning contributes to attitudes toward social partners, and they highlight the importance of considering individual differences in learning strategies during social interactions to understand the effects of rewards on social attitudes and decisions.

### Model-Based and Model-Free Social Cognition

Our central finding – of model-free learning in social impression formation – offers novel theoretical implications for social cognition, learning, and attitudes. First, our findings highlight a role for reward-based reinforcement learning in social interactions. Previous impression formation research demonstrates that people learn about the traits of others in order to predict how others will behave (Heider, 1958). For instance, by observing financial advisors, people can form impressions of an advisor’s competence and predict that advisor’s future performance (Boorman et al., 2013; Leong and Zaki, 2018). Our results introduce a complementary mode of social learning based on reward: people also learn whom to choose and whom to like through instrumental learning, such as directly choosing an advisor and experiencing rewards as a result.

The observation of model-free social learning, in particular, supports the proposed role of habit in social cognition. In model-free learning, people repeat previously-rewarded choices in a relatively inflexible manner – the hallmark of a habit. Habits may therefore influence social behavior: because habits reflect routinized responses that operate most adaptively in invariable environments, they may fill in the gaps between goal-directed responses to facilitate social behavior. In some cases, habits may have harmful effects; for example, people may persist in interacting with social partners with whom they had positive past experiences, even when other partners might be equally or more relevant to one’s current goals. In other cases, habits may be beneficial, leading an individual to approach a previously-rewarding person while distracted by their pursuit of an unrelated goal – perhaps eliciting help, if needed, or simply avoiding a social faux pas. In both cases, their effects may be subtle, relative to goal-directed responses, yet still crucial to adaptive social function.

Although model-based and model-free learning offer different benefits and costs, their concerted function may promote successful social interactions. Social life offers a wealth of information about other people – their traits, preferences, and emotions – which lets us know whom to interact with and how to interact with them. Through experience, we learn which members of our social networks to turn to for empathy as opposed to fun (Morelli et al., 2017) and which verbal or facial cues predict different emotions for close others (Zaki et al., 2016). Model-free learning offers a computationally simple way to learn how to act around others given this wealth of information, requiring little deliberation (Otto et al., 2013). Yet, at the same time, model-free learning is relatively inflexible, leaving people unable to adapt as contingencies change or to plan ahead in novel settings. By comparison, model-based learning requires greater effort but allows people to adapt to new contingencies and make novel plans – for instance, choosing a gift for another person for the first time given knowledge about their preferences. Both types of learning are functional, with tradeoffs that depend on the particulars of a situation, and thus an important goal of future research will be to explore how these tradeoffs are managed and prioritized across situations.

It is notable that participants’ behavior was highly model-based in our study, on average – more so than in past work using this task (Kool et al., 2017; see also Da Silva and Hare, 2019). It is possible that the social framing of the task made it easier for people to reason in a model-based manner, much as people find it easier to reason about social relations than non-social relations (Cosmides, 1989; Mason et al., 2010). Moreover, our instructions framed rewards in terms of stock performance, which offers a familiar and intuitive explanation for drifting outcomes. While it is possible that these features made our instructions clearer relative to past work (Da Silva and Hare, 2019), the familiarity of concepts used in our task framing may have facilitated model-based choices – an interesting possibility for future research.

Finally, and more broadly, this work sheds light on how multiple forms of learning and memory can contribute to social cognition. Based on research in cognitive neuroscience (Squire, 2004; Henke, 2010), Amodio (2019; see also Amodio and Ratner, 2011) theorized that social cognition comprises multiple distinct and interactive learning and memory systems, including habits. Although classic work in social psychology has focused primarily on the roles of conceptual associations and Pavlovian forms of learning, research has just recently begun to probe the role of reward-based forms of learning in social cognition (Hackel et al., 2015; Lindström and Tobler, 2018). To date, these studies have not distinguished between types of computations that may underlie instrumental learning from rewards. Here, by using a two-step learning task to examine social learning, we were able to dissociate model-based and model-free forms of reward learning and, in doing so, provide new evidence for the role of multiple learning systems, functioning in concert, in social cognition.

### Potential Limitations

The goal of this research was to examine learning processes that give rise to habitual behavior. However, there remain open questions about the extent to which model-free learning, as assessed in sequential decision-making (i.e., two-step) tasks, corresponds to traditional definitions of habit. First, questions have been raised as to whether additional strategies may contribute to observed effects of model-free learning in sequential decision tasks (Dezfouli and Balleine, 2012; Da Silva and Hare, 2019; but see Morris and Cushman, 2019), just as other representations may contribute to observed effects of model-based learning (Momennejad et al., 2017; Russek et al., 2017).

Although our task was designed to examine two specific learning processes, it is useful to consider the possibility of alternative ways of representing the task and outcomes that might yield different inferences. For instance, if participants grouped the two “Axiom” advisors under one abstract action representation of “pro-Axiom-choice” (possibly through model-based processes), then putative patterns of model-based learning might actually reflect model-free learning over such groupings; conversely, if participants represented four end states in the task – acting as if there were two distinct Axiom stocks and two distinct Zephyr stocks depending on the advisor chosen – then putative patterns of model-free learning could reflect model-based learning. However, we believe such a four-state task representation is unlikely, given that the instructions and visual display emphasized that there were two end states, each reached from two advisors. For a participant to use a 4-state task representation, they would have to ignore this information and the actual transition structure of the task, associating end-states with actions used to get there, in which case it may not be obvious that this would still be a model-based controller (see Morris and Cushman, 2019, for related discussion). Future work could test whether people generate unexpected task representations and whether these contribute to learning.

More broadly, people may use learning and choice strategies not encapsulated by our task and analyses, moving beyond the two approaches studied here (see Supplementary Material for further discussion). For instance, in other settings, people might choose individual advisors based on trait impressions (Hackel et al., 2015) or might learn specific motor actions (Shahar et al., 2019) – such as pushing a particular button or walking toward a colleague’s office – in addition to learning the value of a social partners. Although our experiment did not address these broader theoretical questions regarding model-based and model-free learning accounts, future research on reinforcement learning in social cognition will benefit from advances in our understanding of these processes as they develop.

Second, there is some debate on whether – and to what extent – model-free learning maps on to traditional definitions of habitual control (Miller et al., 2019; see also Gillan et al., 2015; Sjoerds et al., 2016). Miller et al. (2019) argue that traditional conceptualizations of habits reflect stimulus-response associations devoid of expected value representations (i.e., are *value-free*), whereas model-free algorithms still depend on the expected value representations associated with a learner’s available actions (i.e., are *value-based*). In this view, habits form directly through action repetition within a given context, regardless of reward outcomes. It is possible that both model-free RL and action repetition contribute to behaviors commonly considered habitual (Pauli et al., 2018). These processes might align with a theoretical distinction between “direct” cuing of habits, in which responses are directly associated with context cues, and “motivated” cuing of habits, in which responses depend on the motivation linked to a behavior through past rewards (Wood and Neal, 2007). To complement and extend our findings, future work could consider these varied approaches.

### New Questions About Habits in Social Behavior

Our use of the two-step task to probe the role of habits in social cognition raises several new questions regarding other aspects of habits in social life. For instance, a classic marker of a habit is its persistence even when it no longer fulfills a valued goal (Wood and Rünger, 2016). Past work suggests that reward feedback in social interaction can have such a persistent impact (Hackel et al., 2015). Future work should consider tasks traditionally employed to test for this kind of habitual persistence, such as the slips-of-action paradigm (e.g., Gillan et al., 2011; de Wit et al., 2012) or outcome devaluation/revaluation procedures (e.g., Valentin et al., 2007; de Wit et al., 2009; Tricomi et al., 2009; see Foerde, 2018, for review).

Our findings raise further questions regarding the specificity of habits in social impressions, relationships, and behaviors. For example, do people form habits to interact with specific partners in specific contexts? Or do they form habits to approach or avoid social interaction in general? Are there benefits to forming such social habits? Answering these questions promises to illuminate the structure of people’s social lives, much as advances in habit research sheds light on how habits can promote healthy eating, exercising, or studying (Galla and Duckworth, 2015; Lin et al., 2016).

Finally, the implications of our findings extend to other areas of research within social psychology, such as intergroup relations, complementing recent work suggesting that model-free learning may underlie implicit attitudes toward social groups (Kurdi et al., 2019). The concept of habit has previously been invoked in prior theories of social attitudes, such as to describe the phenomenon of implicit prejudice and the difficulty people have in ridding themselves of it (e.g., “breaking the prejudice habit,” Devine, 1989; Devine et al., 2012). However, this usage has been largely colloquial or metaphorical, as previous research has not used methods capable of assessing habit-like patterns of preference and choice. Our findings suggest that social experiences may indeed give rise to a form of habit, but these are rooted more directly in reward-based action tendencies than in conceptual processes such as stereotypes.

Nevertheless, if some aspects of prejudice are truly habit-like, then they may be extraordinarily difficult to control or eradicate. As such, interventions involving the replacement of a biased thought or action with an egalitarian response (Devine, 1989) or changes in the situational affordances for bias expression (Amodio and Swencionis, 2018) should be more effective than methods for unlearning bias (Lai et al., 2014). Furthermore, an intervention aimed at “unlearning” a habit-like response would require action-based interventions, in contrast to conventional interventions aimed at modifying a person’s beliefs and values. As our conceptualization of habits in social cognition develops, it may begin to elucidate psychological processes in other domains as well.

## Conclusion

Habits are integral to everyday human behavior, and they may also support our social behaviors. Our findings represent an initial demonstration that habit-like learning processes are also involved in the formation of social preferences and attitudes. These findings expand our understanding of how learning and memory systems support social cognition and provide a foundation for new research on the role of habit in social learning.

## Data Availability Statement

The datasets generated for this study are available on request to the corresponding author.

## Ethics Statement

The studies involving human participants were reviewed and approved by New York University Committee on Activities Involving Human Subjects. The patients/participants provided their written informed consent to participate in this study.

## Author Contributions

All authors developed the theoretical ideas, questions, and approach. LH and JB designed the task. JB collected the data. LH and BL analyzed the data. LH and DA drafted the manuscript, with input and edits by JB and BL.

## Conflict of Interest

The authors declare that the research was conducted in the absence of any commercial or financial relationships that could be construed as a potential conflict of interest.

## Footnotes

1 In contrast, we did not observe an interaction between the w parameter and model-based values (see Supplementary Material). This finding is consistent with the fact that model-based learning was relatively high across participants, whereas not all participants showed a meaningful degree of model-free learning (i.e., w < 1).

## References

[B1] Amodio D. M. (2019). Social Cognition 2.0: an interactive memory systems account. *Trends Cogn. Sci.* 23 21–33. 10.1016/j.tics.2018.10.002 30466793

[B2] Amodio D. M. Berg J. J. (2018). Toward a multiple memory systems model of attitudes and social cognition. *Psychol. Inq.* 29 14–19. 10.1080/1047840x.2018.1435620

[B3] Amodio D. M. Ratner K. G. (2011). A memory systems model of implicit social cognition. *Curr. Dir. Psychol. Sci.* 20 143–148. 10.1177/0963721411408562

[B4] Amodio D. M. Swencionis J. K. (2018). Proactive control of implicit bias: a theoretical model and implications for behavior change. *J. Personal. Soc. Psychol.* 115 255–275. 10.1037/pspi0000128 30024242

[B5] Asch S. E. (1946). Forming impressions of personality. *J. Abnorm. Soc. Psychol.* 41 258–290. 10.1037/h0055756 20995551

[B6] Balleine B. W. Dickinson A. (1998). Goal-directed instrumental action: contingency and incentive learning and their cortical substrates. *Neuropharmacology* 37 407–419. 10.1016/s0028-3908(98)00033-1 9704982

[B7] Bargh J. A. Ferguson M. J. (2000). Beyond behaviorism: on the automaticity of higher mental processes. *Psychol. Bull.* 126 925–945. 10.1037//0033-2909.126.6.925 11107883

[B8] Bates D. Mächler M. Bolker B. Walker S. (2015). Fitting linear mixed-effects models using lme4. *J. Stat. Softw.* 67 1–48.

[B9] Boorman E. D. O’Doherty J. P. Adolphs R. Rangel A. (2013). The behavioral and neural mechanisms underlying the tracking of expertise. *Neuron* 80 1558–1571. 10.1016/j.neuron.2013.10.024 24360551 PMC3878380

[B10] Brewer M. B. (1988). “A dual process model of impression formation,” in *Advances in Social Cognition*, Vol. 1 eds Srull T. K. Wyer R. S. Jr. (Hillsdale, NJ: Lawrence Erlbaum Associates), 1–36.

[B11] Carlston D. E. Skowronski J. J. (1994). Savings in the relearning of trait information as evidence for spontaneous inference generation. *J. Personal. Soc. Psychol.* 66 840–856. 10.1037//0022-3514.66.5.840

[B12] Cosmides L. (1989). The logic of social exchange: has natural selection shaped how humans reason? Studies with the Wason selection task. *Cognition* 31 187–276. 10.1016/0010-0277(89)90023-1 2743748

[B13] Da Silva C. F. Hare T. A. (2019). Humans are primarily model-based and not model-free learners in the two-stage task. *BioRxiv.* [preprint]. 10.1101/682922

[B14] Daw N. D. Gershman S. J. Seymour B. Dayan P. Dolan R. J. (2011). Model-based influences on humans’ choices and striatal prediction errors. *Neuron* 69 1204–1215. 10.1016/j.neuron.2011.02.027 21435563 PMC3077926

[B15] De Houwer J. Hendrickx H. Baeyens F. (1997). Evaluative learning with “subliminally” presented stimuli. *Conscious. Cogn.* 6 87–107. 10.1006/ccog.1996.0281 9170563

[B16] de Wit S. Corlett P. R. Aitken M. R. Dickinson A. Fletcher P. C. (2009). Differential engagement of the ventromedial prefrontal cortex by goal-directed and habitual behavior toward food pictures in humans. *J. Neurosci.* 29 11330–11338. 10.1523/JNEUROSCI.1639-09.2009 19741139 PMC3443853

[B17] de Wit S. Watson P. Harsay H. A. Cohen M. X. van de Vijver I. Ridderinkhof K. R. (2012). Corticostriatal connectivity underlies individual differences in the balance between habitual and goal-directed action control. *J. Neurosci.* 32 12066–12075. 10.1523/JNEUROSCI.1088-12.2012 22933790 PMC6621537

[B18] Devine P. G. (1989). Stereotypes and prejudice: their automatic and controlled components. *J. Personal. Soc. Psychol.* 56 5–18. 10.1037//0022-3514.56.1.5

[B19] Devine P. G. Forscher P. S. Austin A. J. Cox W. T. (2012). Long-term reduction in implicit race bias: a prejudice habit-breaking intervention. *J. Exp. Soc. Psychol.* 48 1267–1278. 10.1016/j.jesp.2012.06.003 23524616 PMC3603687

[B20] Dezfouli A. Balleine B. W. (2012). Habits, action sequences and reinforcement learning. *Eur. J, Neurosci.* 35 1036–1051. 10.1111/j.1460-9568.2012.08050.x 22487034 PMC3325518

[B21] Dickinson A. Balleine B. (1994). Motivational control of goal-directed action. *Anim. Learni. Behav.* 22 1–18. 10.3758/bf03199951

[B22] Doll B. B. Duncan K. D. Simon D. A. Shohamy D. Daw N. D. (2015). Model-based choices involve prospective neural activity. *Nat. Neurosci.* 18 767–772. 10.1038/nn.3981 25799041 PMC4414826

[B23] Fiske S. T. Neuberg S. L. (1990). “A continuum of impression formation, from category-based to individuating processes: Influences of information and motivation on attention and interpretation,” in *Advances in Experimental Social Psychology*, Vol. 23 ed. Zanna M. P., (New York, NY: Academic Press), 1–74. 10.1016/s0065-2601(08)60317-2

[B24] Fitzsimons G. M. Anderson J. (2013). “Interpersonal cognition: seeking, understanding, and maintaining relationships,” in *Handbook of Social Cognition*, ed. Carlston D., (New York, NY: Oxford University Press), 590–615.

[B25] Foerde K. (2018). What are habits and do they depend on the striatum? A view from the study of neuropsychological populations. *Curr. Opin. Behav. Sci.* 20 17–24. 10.1016/j.cobeha.2017.08.011

[B26] Galla B. M. Duckworth A. L. (2015). More than resisting temptation: beneficial habits mediate the relationship between self-control and positive life outcomes. *J. Personal. Soc. Psychol.* 109 508–525. 10.1037/pspp0000026 25643222 PMC4731333

[B27] Gershman S. J. (2016). Empirical priors for reinforcement learning models. *J. Math. Psychol.* 71 1–6. 10.1016/j.jmp.2016.01.006

[B28] Gillan C. M. Otto A. R. Phelps E. A. Daw N. D. (2015). Model-based learning protects against forming habits. *Cogn. Affect. Behav. Neurosci.* 15 523–536. 10.3758/s13415-015-0347-6 25801925 PMC4526597

[B29] Gillan C. M. Papmeyer M. Morein-Zamir S. Sahakian B. J. Fineberg N. A. Robbins T. W. (2011). Disruption in the balance between goal-directed behavior and habit learning in obsessive-compulsive disorder. *Am. J. Psychiatry* 168 718–726. 10.1176/appi.ajp.2011.10071062 21572165 PMC3533260

[B30] Gureckis T. M. Martin J. McDonnell J. Rich A. S. Markant D. Coenen A. (2016). psiTurk: an open-source framework for conducting replicable behavioral experiments online. *Behav. Res. Methods* 48 829–842. 10.3758/s13428-015-0642-8 26428910

[B31] Hackel L. M. Amodio D. M. (2018). Computational neuroscience approaches to social cognition. *Curr. Opin. Psychol.* 24 92–97. 10.1016/j.copsyc.2018.09.001 30388495

[B32] Hackel L. M. Doll B. B. Amodio D. M. (2015). Instrumental learning of traits versus rewards: dissociable neural correlates and effects on choice. *Nat. Neurosci.* 18 1233–1235. 10.1038/nn.4080 26237363

[B33] Heider F. (1958). *The Psychology of Interpersonal Relations.* New York, NY: Wiley.

[B34] Henke K. (2010). A model for memory systems based on processing modes rather than consciousness. *Nat. Rev. Neurosci.* 11 523–532. 10.1038/nrn2850 20531422

[B35] Hofmann W. De Houwer J. Perugini M. Baeyens F. Crombez G. (2010). Evaluative conditioning in humans: a meta-analysis. *Psychol. Bull.* 136 390–421. 10.1037/a0018916 20438144

[B36] Jones E. E. Davis K. E. (1965). “From acts to dispositions: The attribution process in person perception,” in *Advances in Experimental Social Psychology*, Vol. 2 ed. Berkowitz L., (New York, NY: Academic Press), 219–266. 10.1016/s0065-2601(08)60107-0

[B37] Jones R. M. Somerville L. H. Li J. Ruberry E. J. Libby V. Glover G. (2011). Behavioral and neural properties of social reinforcement learning. *J. Neurosci.* 31 13039–13045. 21917787 10.1523/JNEUROSCI.2972-11.2011 PMC3303166

[B38] Kool W. Cushman F. A. Gershman S. J. (2016). When does model-based control pay off? *PLoS Comput. Biol.* 12:e1005090. 10.1371/journal.pcbi.1005090 27564094 PMC5001643

[B39] Kool W. Gershman S. J. Cushman F. A. (2017). Cost-benefit arbitration between multiple reinforcement-learning systems. *Psychol. Sci.* 28 1321–1333. 10.1177/0956797617708288 28731839

[B40] Kurdi B. Gershman S. J. Banaji M. R. (2019). Model-free and model-based learning processes in the updating of explicit and implicit evaluations. *Proc. Natl. Acad. Sci. U.S.A.* 116 6035–6044. 10.1073/pnas.1820238116 30862738 PMC6442571

[B41] Kuznetsova A. Brockhoff P. B. Christensen R. H. B. (2016). *lmerTest: Tests for Random and Fixed Effects for Linear Mixed Effect Models (Lmer Objects of Lme4 Package). R package (Version 2.0–32).*

[B42] Lai C. K. Marini M. Lehr S. A. Cerruti C. Shin J. E. L. Joy-Gaba J. A. (2014). Reducing implicit racial preferences: I. A comparative investigation of 17 interventions. *J. Exp. Psychol.: Gen*, 143 1765–1785. 10.1037/a0036260 24661055

[B43] Leong Y. C. Zaki J. (2018). Unrealistic optimism in advice taking: a computational account. *J. Exp. Psychol.* 147 170–189. 10.1037/xge0000382 29154614

[B44] Lin A. Adolphs R. Rangel A. (2011). Social and monetary reward learning engage overlapping neural substrates. *Soc. Cogn. Affect. Neurosci.* 7 274–281. 10.1093/scan/nsr006 21427193 PMC3304477

[B45] Lin P. Y. Wood W. Monterosso J. (2016). Healthy eating habits protect against temptations. *Appetite* 103 432–440. 10.1016/j.appet.2015.11.011 26585633

[B46] Lindström B. Selbing I. Molapour T. Olsson A. (2014). Racial bias shapes social reinforcement learning. *Psychol. Sci.* 25 711–719. 10.1177/0956797613514093 24458270

[B47] Lindström B. Tobler P. N. (2018). Incidental ostracism emerges from simple learning mechanisms. *Nat. Hum. Behav.* 2 405–414. 10.1038/s41562-018-0355-y 31024161

[B48] Mason M. F. Magee J. C. Kuwabara K. Nind L. (2010). Specialization in relational reasoning: the efficiency, accuracy, and neural substrates of social versus nonsocial inferences. *Soc. Psychol. Personal. Sci.* 1 318–326. 10.1177/1948550610366166

[B49] Miller K. J. Shenhav A. Ludvig E. A. (2019). Habits without values. *Psychol. Rev.* 126 292–311. 10.1037/rev0000120 30676040 PMC6548181

[B50] Momennejad I. Russek E. M. Cheong J. H. Botvinick M. M. Daw N. D. Gershman S. J. (2017). The successor representation in human reinforcement learning. *Nat. Hum. Behav.* 1 680–692. 10.1038/s41562-017-0180-8 31024137 PMC6941356

[B51] Morelli S. A. Ong D. C. Makati R. Jackson M. O. Zaki J. (2017). Empathy and well-being correlate with centrality in different social networks. *Proc. Natl. Acad. Sci. U.S.A.* 114 9843–9847. 10.1073/pnas.1702155114 28851835 PMC5604000

[B52] Morey R. D. (2008). Confidence intervals from normalized data: a correction to cousineau (2005). *Tutor. Quant. Methods Psychol.* 4 61–64. 10.20982/tqmp.04.2.p061

[B53] Morris A. Cushman F. A. (2019). Model-free RL or action sequences? *PsyArXiv.* [preprint]. 10.31234/osf.io/k67tm PMC6933525 31920900

[B54] Moskowitz G. B. Roman R. J. (1992). Spontaneous trait inferences as self-generated primes: implications for conscious social judgment. *J. Personal. Soc. Psychol.* 62 728–738. 10.1037//0022-3514.62.5.728 1593417

[B55] Olson M. A. Fazio R. H. (2006). Reducing automatically activated racial prejudice through implicit evaluative conditioning. *Personal. Soc. Psychol. Bull.* 32 421–433. 10.1177/0146167205284004 16513796

[B56] Otto A. R. Gershman S. J. Markman A. B. Daw N. D. (2013). The curse of planning: dissecting multiple reinforcement-learning systems by taxing the central executive. *Psychol. Sci.* 24 751–761. 10.1177/0956797612463080 23558545 PMC3843765

[B57] Pauli W. M. Cockburn J. Pool E. R. Pérez O. D. O’Doherty J. P. (2018). Computational approaches to habits in a model-free world. *Curr. Opin. Behav. Sci.* 20 104–109.

[B58] R Core Team, (2016). *R: A Language and Environment for Statistical Computing (Version 3.3.1).* vienna: R Core Team.

[B59] Robbins T. W. Costa R. M. (2017). Habits. *Curr. Biol.* 27 R1200–R1206. 10.1016/j.cub.2017.09.060 29161553

[B60] Russek E. M. Momennejad I. Botvinick M. M. Gershman S. J. Daw N. D. (2017). Predictive representations can link model-based reinforcement learning to model-free mechanisms. *PLoS Comput. Biol.* 13:e1005768. 10.1371/journal.pcbi.1005768 28945743 PMC5628940

[B61] Rydell R. J. McConnell A. R. (2006). Understanding implicit and explicit attitude change: a systems of reasoning analysis. *J. Personal. Soc. Psychol.* 91 995–1008. 10.1037/0022-3514.91.6.995 17144760

[B62] Shahar N. Moran R. Hauser T. U. Kievit R. A. McNamee D. Moutoussis M. (2019). Credit assignment to state-independent task representations and its relationship with model-based decision making. *Proc. Natl. Acad. Sci. U.S.A.* 116 15871–15876. 10.1073/pnas.1821647116 31320592 PMC6689934

[B63] Sjoerds Z. Dietrich A. Deserno L. De Wit S. Villringer A. Heinze H. J. (2016). Slips of action and sequential decisions: a cross-validation study of tasks assessing habitual and goal-directed action control. *Front. Behav. Neurosci.* 10:234. 10.3389/fnbeh.2016.00234 28066200 PMC5167743

[B64] Squire L. R. (2004). Memory systems of the brain: a brief history and current perspective. *Neurobiol. Learn. Mem.* 82 171–177. 10.1016/j.nlm.2004.06.005 15464402

[B65] Sutton R. S. Barto A. G. (1998). *Introduction to Reinforcement Learning.* Cambridge, MA: MIT Press.

[B66] Sweldens S. Corneille O. Yzerbyt V. (2014). The role of awareness in attitude formation through evaluative conditioning. *Personal. Soc. Psychol. Rev.* 18 187–209. 10.1177/1088868314527832 24669003

[B67] Tamir D. I. Thornton M. A. (2018). Modeling the predictive social mind. *Trends Cogn. Sci.* 22 201–212. 10.1016/j.tics.2017.12.005 29361382 PMC5828990

[B68] Thorndike E. (1911). *Animal Intelligence.* New York, NY: Hafner. 10.1126/science.7.181.818 17769765

[B69] Tricomi E. Balleine B. W. O’Doherty J. P. (2009). A specific role for posterior dorsolateral striatum in human habit learning. *Eur. J. Neurosci.* 29 2225–2232. 10.1111/j.1460-9568.2009.06796.x 19490086 PMC2758609

[B70] Uleman J. S. Kressel L. M. (2013). “A brief history of theory and research on impression formation,” in *Oxford Handbook of Social Cognition*, ed. Carlston D. E., (New York, NY: Oxford University Press), 53–73.

[B71] Valentin V. V. Dickinson A. O’Doherty J. P. (2007). Determining the neural substrates of goal-directed learning in the human brain. *J. Neurosci.* 27 4019–4026. 10.1523/jneurosci.0564-07.2007 17428979 PMC6672546

[B72] Walther E. (2002). Guilty by mere association: evaluative conditioning and the spreading attitude effect. *J. Persona. Soc. Psychol.* 82 919–934. 10.1037//0022-3514.82.6.919 12051580

[B73] Winter L. Uleman J. S. (1984). When are social judgments made? Evidence for the spontaneousness of trait inferences. *J. Personal. Soc. Psychol.* 47 237–252. 10.1037//0022-3514.47.2.237 6481615

[B74] Wood W. (2017). Habit in personality and social psychology. *Personal. Soc. Psychol. Rev.* 21 389–403. 10.1177/1088868317720362 28737111

[B75] Wood W. Neal D. T. (2007). A new look at habits and the habit-goal interface. *Psychol. Rev.* 114 843–863. 10.1037/0033-295x.114.4.843 17907866

[B76] Wood W. Rünger D. (2016). Psychology of habit. *Annu. Rev. Psychol.* 67 289–314. 26361052 10.1146/annurev-psych-122414-033417

[B77] Wyer R. S. Jr. Carlston D. E. (1979). *Social Cognition, Inference, and Attribution.* Hillsdale, NJ: Erlbaum Publishers.

[B78] Zaki J. Kallman S. Wimmer G. E. Ochsner K. Shohamy D. (2016). Social cognition as reinforcement learning: feedback modulates emotion inference. *J. Cogn. Neurosci.* 28 1270–1282. 10.1162/jocn_a_00978 27167401

