# Supplementary material for: Model-Based and Model-Free Social Cognition: Investigating the Role of Habit in Social Attitude Formation and Choice
Source: Front Psychol. 2019 Nov 21;10:2592. doi: 10.3389/fpsyg.2019.02592 (PMC6881302; doi:10.3389/fpsyg.2019.02592)
Supplement: Supplementary file 1 [file Table_1.docx]

**Supplemental Materials**

**Supplemental Methods**

**Computational Model.** We fit behavior to a computational model described in prior work (Kool, Gershman, & Cushman, 2017). This model and its theoretical background have been described extensively in previous work; here, we briefly outline its instantiation in the present research.

The model assumes that participants employ a hybrid of model-based and model-free learning. At the second stage of a trial in the two-step task (i.e., revealing the stock payoff), model-based and model-free values are equivalent, given that there are no further state transitions (e.g., additional stimuli) following second-stage rewards. As a result, for each second-state *S2,* state-action values *Q2* are learned for each action *a2* (i.e., selecting the stock to reveal a payout). These values are updated on each trial *t* after rewards are received, according to:

$$Q2\left( {s2}_{t},{a2}_{t} \right)=Q2\left( {s2}_{t},{a2}_{t} \right)+\alpha\delta_{2,t}$$

where $\delta_{2,t}$ is the prediction error at the second stage:

$$\delta_{2,t}=r_{t}-Q2\left( {s2}_{t},{a2}_{t} \right)$$

given reward *r* and learning rate $\alpha$, which controls the impact of new rewards on values.

At the first stage, model-based and model-free values differ. The model-free system uses SARSA(λ) (state-action-reward-state-action temporal difference learning algorithm with eligibility trace parameter λ) to learn a value, *QMF*, for each action *a1* at each first stage *s1* (i.e., values for choosing each advisor). These values are updated after each stage. Following the first stage, no direct rewards are experienced, and so model-free values for advisors are updated based on the value of the second stage action:

$$QMF\left( {s1}_{t},{a1}_{t} \right)=QMF\left( {s1}_{t},{a1}_{t} \right)+\alpha\delta_{1,t}$$

where

$$\delta_{1,t}=Q2\left( {s2}_{t},{a2}_{t} \right)-QMF\left( {s1}_{t},{a1}_{t} \right)$$

Following the second stage action, model-free values for advisors are updated based on the rewards experienced:

$$QMF\left( {s1}_{t},{a1}_{t} \right)=QMF\left( {s1}_{t},{a1}_{t} \right)+\lambda\alpha\delta_{2,t}$$

where $\lambda$ is an eligibility trace parameter that down-weights the impact of the second-stage prediction error on first-stage values. Eligibility traces are a key component of classic model-free RL algorithms (Sutton & Barto, 1998), requiring only a memory of what states have recently been visited; they do not require a model of transitions.

The model-based system uses the task transition structure to choose an advisor at Stage 1, planning ahead based on the *Q2* values of each stock. Formally, this depends on the probability of transitioning to each second-stage state and the reward available for the best action in each second-stage state (see Kool et al., 2017; Doll et al., 2015). Since transitions in the current task were deterministic and there was only one action available at the second stage, this simplifies in the present task to:

$$Q_{MB}\left( {s1}_{t},{a1}_{t} \right)=Q2(S\left( {s1}_{t},{a1}_{t} \right),A\left( {s1}_{t},{a1}_{t} \right))$$

where$S\left( {s1}_{t},{a1}_{t} \right)$ is the second-stage state that would be produced by choosing action *a1* in the first-stage state *s1* and $A\left( {s1}_{t},{a1}_{t} \right)$ is the action that would be available in that state. (Note that only one action was available in each second state; one stock was shown onscreen, and participants pressed a key to reveal the payout.)

To make a choice at the first stage, the model combines model-based and model-free values using a weighting parameter *w:*

$$Q_{net}\left( {s1}_{t},{a1}_{t} \right)=wQ_{MB}\left( {s1}_{t},{a1}_{t} \right)+\left( 1-w \right)Q_{MF}({s1}_{t},{a1}_{t})$$

Note that if *w* = 0, choices would be fully model-free, and if *w* = 1, choices would be fully model-based.

Finally, choices were modeled using a softmax choice function:

$$P\left( a_{i,t}=a | s_{i,t} \right)=\frac{exp(\beta\left[ Q_{net}\left( s_{i,t},a \right)+\pi\cdot rep\left( a \right)+\rho\cdot resp\left( a \right) \right])}{\sum_{a^{'}} exp(\beta\left[ Q_{net}\left( s_{i,t},a^{'} \right)+\pi\cdot rep\left( a^{'} \right)+\rho\cdot resp\left( a^{'} \right) \right])}$$

where *β* is an inverse temperature parameter controlling the stochasticity of choice, *rep(a)* indicates if first-stage action *a* is the same one chosen on the previous trial (1 if yes, 0 if no), *resp(a)* indicates if the same response key was pressed as on the previous trial (1 if yes, 0 if no), and *π* and *ρ* are “stickiness” and “response stickiness” parameters that weight these indicators, respectively. The “stickiness” terms control the extent to which participants show perseveration in choosing the same stimulus (i.e., the same advisor) and perseveration in making the same button press (e.g., pressing the button on the right). By accounting for “stickiness” in choice, the model can account for variance in behavior related to value-free choice repetition, allowing sharper estimates of model-based and model-free learning.

**Model-fitting.** Parameters were estimated using maximum a posteriori (MAP) estimation with empirical priors (Gershman, 2016). Following Kool et al. (2017), weak priors were used for the inverse temperature parameter, *β ~* Gamma(4.82, 0.88), and stickiness parameters, *π,* *ρ ~* (0.15, 1.42); other parameters had flat priors. The learning rate, weighting parameter, and eligibility trace decay were bounded between 0 and 1; the inverse temperature parameter was bounded between 0 and 20; and the stickiness parameters were bounded between -20 and 20.


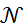

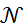


**Model comparison.** Our parameter fits revealed a high degree of model-based learning (Table S1); indeed, 20 participants out of 65 had fitted *w* parameters equal to 1. As a complementary approach to test the impact of both model-free and model-based learning, we fit an alternate model in which the *w* parameter was constrained to 1 (fully model-based) for all subjects. We compared this model to the hybrid model—which contains an additional free parameter—using Bayesian model selection (Stephan, Penny, Daunizeau, & Moran, 2009) as implemented in the m-fit package for Matlab (Gershman, 2016). Surprisingly, this procedure supported the constrained model (*w* = 1), exceedance probability = 1.

Given that the regression analysis showed positive evidence of model-free learning—an interaction of Reward × Start State that would not be produced by model-based learning alone—it is possible that the model comparison failed to select the hybrid model given high *w* parameters on average. Indeed, a pattern of qualitative behavioral results that is predicted by one model but not another may provide stronger evidence than quantitative computational model fits (Palminteri, Wyart, & Koechlin, 2017). Nonetheless, to explore this possibility, we conducted a series of simulations. We simulated the task 100 times using the exact parameter fits from our subjects, with each simulated subject’s parameters corresponding to one real subject’s parameters; we next simulated the task 100 times using the same parameter fits but with the *w* parameter fixed to 1 for each subject. For each simulation, we conducted the regression and model comparison analyses on the simulated data. This procedure allowed to ask how well each analysis detected effects under each ground truth (*w* varying vs *w* of 1 across the full sample) and allowed us to rule out the possibility that the regression analysis might produce an artifactual model-free effect even when *w* = 1 in all subjects.

When *w* was set to 1 for all subjects, the regression analysis returned a significant model-free effect in only 5% of simulations, consistent with an alpha rate of .05. Moreover, the model comparison procedure supported the constrained model (*w* = 1) in all iterations. This simulation indicates that the regression analysis would not artifactually produce evidence of model-free learning if the true *w* were equal to 1 in all subjects.

When *w* was high but less than 1 in most subjects (using the actual fits from our sample), the model-comparison procedure still favored the constrained model in all iterations. This result demonstrates that model comparison may fail to select the hybrid model if the *w* parameter is quite high on average. However, the regression analysis recovered a significant model-free effect in 53% of simulations, suggesting that this analysis can correctly recover a model-free effect, although it was somewhat underpowered to do so.

Altogether, these simulations support the conclusions in the main text: the regression analysis revealed the influence of model-free learning in choice on average, although it is possible that this effect was not present in each individual subject.

**Generative model for plots.** To generate the model predictions shown in Figure 2, we simulated the task 100 times with 65 subjects given the model described above. To do so without drawing on our own data, we used the median parameter fits reported by Kool et al. (2017), changing only the *w* parameter to 0 or 1 to simulate fully model-free or model-based behavior, respectively.

**Supplemental Results**

After the main task, participants estimated the average number of points paid out on each trial by each stock over the course of the task. This provided another opportunity to check that participants successfully learned about the task contingencies. We therefore fit a regression predicting these estimates as a function of the actual average number of points paid out by each stock. Given that each participant made two ratings (one for each stock), we used mixed effects linear regression with random effects for the intercept and slope. This analysis revealed that participant estimates were strongly calibrated to actual point averages, *b* = 1.54, *SE* = 0.26, *t*(39.96) = 5.80, *p* < .001. (Note that we did not expect these point estimates to vary based on a participant’s degree of model-based or model-free learning; instead, model-based and model-free learners would only differ in how they use estimates of a stock’s value during choice. Indeed, in our model, the value of each stock was updated with a model-free update in the second stage of the task.)

**Supplemental Discussion**

Our analyses focused on model-based learning and model-free learning, revealed in the present task by the extent to which participants repeated choices of rewarding stocks regardless of seeing the same or a different advisor (model-based) or repeated choices of specific rewarding advisors (model-free). However, it is worth noting other learning and choice processes and other cognitive representations that could give rise to behavior in this task and in other settings.

**Impression Formation**

In the present task, model-based learners would track the value of stock outcomes and choose advisors based on stocks, whereas model-free learners would track the value of advisors. This is because there was no basis in the present task for forming different impressions of two advisors who led to the same stock; as a result, a model-based algorithm would not form distinct value representations for different advisors, whereas a model-free learner would. In other tasks, however, people could track the value of interacting with specific advisors via model-based learning as well. For instance, if provided with unique feedback about each advisor, people might form trait impressions of each advisor’s competence and use these impressions to make choices (Hackel et al., 2015); these choices may be model-based choices rooted in prospection about the outcomes a generous or competent partner is likely to provide. As a result, both model-based impression formation and model-free reward learning might give rise to distinct values of specific social partners in other settings.

**Motor Actions**

We focus in the present work on choices of advisors, but participants also could have represented actions in terms of specific motor actions (e.g., right and left button presses; Shahar et al., 2019). Notably, advisors in our task randomly appeared on the right or left side of the screen; as a result, our analyses were orthogonal to any reinforcement of button presses. Nonetheless, it is possible that participants represented both advisors and sides of screen in their choices. If so, people might form habits not just at the level of partner choice (e.g., which colleague to ask for help) but at the level of specific actions (e.g., which office to walk to).

**Supplemental Notes**

**Task Instructions.** Participants saw the instruction text below across a series of screens.

***Screen 1***

Welcome to the experiment! Today you will be playing a game in which you learn to choose financial advisors and stocks in order to make money. It will take approximately 30 minutes if you pay attention to the instructions.

Please read ALL the instructions VERY CAREFULLY. The instructions are somewhat detailed, but you will need to read them carefully in order to complete the experiment.

If you encounter an error today, please email the requester so you can be compensated.

***Screen 2***

In this game, you will be learning about the choices made by four other Amazon Mechanical Turk workers who participated before you.

Each of the four previous MTurk workers were randomly assigned to one of four avatars, which you can see below.

*[Images appear here.]*

This is a standard procedure in this kind of interactive game.

***Screen 3***

When they played the game, the four previous MTurk workers were assigned to the role of ADVISOR. As Advisors, they were given details about two different STOCKS and had to choose ONE to invest in. Each advisor chose only one stock, and they invested in that stock throughout the entire game. They then earned money based on how well each stock performed. You can see images of the two stocks below.

*[Images appear here.]*

***Screen 4***

As part of a second wave of participants, you will play the game as a CLIENT. As a Client, your job is to decide which Advisor you want to hire.

On each round, you will choose an Advisor to hire for that round of the game. You will earn points depending on how well your chosen Advisor's stock performs during that round (a "dividend").

***Screen 5***

After you choose an Advisor to hire, you will see the stock they invested in. When you see the stock, you will press a button to reveal how well the stock performed (in terms of points), which looks like this:

+2

At the end of the game, these points will be exchanged for a cash bonus.

***Screen 6***

If your chosen Advisor's stock performs very well, you will earn a lot of points:

+9

However, sometimes an Advisor's stock doesn't perform well, and you only earn a few points:

+1

The quality of each stock will change during the game. A stock that was bad at the beginning of the game might start performing well, and a stock that initially pays well might perform poorly later on.

Fluctuations in a stock's performance will happen slowly. So, you have to focus to earn as many points as possible by hiring the Advisor with the best stock at each point in time.

***Screen 7***

At the end of the game, points will be converted into a cash bonus. That is, you will earn money based on the number of points you earn for yourself by hiring Advisors and observing their stock's performance.

Do your best to earn as many points as possible by hiring Advisors with well-performing stocks. That way, you'll earn as much money as possible.

***Screen 8***

Once you see the stock that your hired Advisor invested in, you should press the SPACEBAR to find out how it performed. You will then see how many points you earned.

You're now going to practice a few times. You will see a stock, and you will press the SPACEBAR to see how many points it pays out. Pay close attention to how the stocks perform! The stocks may change in how many points they pay out.

(Later, you will practice choosing Advisor. For now, you will only see the stocks.)

*[Participant completes practice trials in which they press the SPACEBAR to reveal a stock’s payout.]*

***Screen 9***

You may have noticed that this stock started out good, and then became less good over time. The performance of each stock will have its own unique pattern. Even if one stock goes from good to bad, the other stock might perform completely differently! To see this, you are going to observe the performance of the other stock.

*[Participant completes practice trials in which they press the SPACEBAR to reveal the other stock’s payout.]*

***Screen 10***

Now that you know how to reveal a stock's performance, you can learn how to choose Advisors and what stocks they invested in. On each round, you will be choosing between two different Advisors:

*[Images of Pair 1]*

OR

*[Images of Pair 2]*

Sometimes, you'll be choosing between the two Advisors on the top, and sometimes you'll be choosing between the two Advisors on the bottom. You can choose the left Advisor by pressing the 'F' key and the right Advisor by pressing the 'J' key.

Please note that the Advisors might switch sides of screen. So pay close attention to the Advisor that you are choosing.

For each pair, one Advisor will have invested in one stock and the other Advisor will have invested in the other stock. Try to choose the Advisor in each pair that you think invested in the stock that is performing better during that round.

***Screen 11***

Now let's practice choosing Advisors to get to certain stocks. First, try to pick the Advisors that invested in the Axiom stock.

(Remember: You can choose the left Advisor by pressing the 'F' key and the right Advisor by pressing the 'J' key.)

*Participant completes practice trials in which they are shown pairs of Advisors and must learn to choose the Advisors that invested in the Axiom stock.*

***Screen 12***

Very good! Now, try to pick the Advisors that invested in the Zephyr stock:

*Participant completes practice trials in which they are shown pairs of Advisors and must learn to choose the Advisors that invested in the Zephyr stock.*

***Screen 13***

You now know how to reveal a stock's performance, as well as which Advisors invested in which stocks, so let's put them together.

Each time, you will (1) pick an Advisor to hire, (2) see the stock that they invested in, and (3) reveal the stock's performance.

Your goal is to earn as many points as possible by choosing Advisors who invested in stocks that are performing well.

Remember that each Advisor only picked ONE stock at the beginning of their study. They DID NOT change their chosen stocks at any point. Therefore, choosing an Advisor will always lead to the same stock.

***Screen 14***

Hint #1: Remember which stocks are performing well. Changes in stock performance happen slowly, so a stock performing well right now will probably still be performing well in the near future.

Hint #2: The Advisor you choose is important because often one stock is performing better than the other stock. So you will want to try to choose the Advisor who invested in the better stock at that time. You will earn more points, and receive a larger cash bonus, by hiring the Advisor that invested in the right stock.

Hint 3: Remember, each stock went up or down on its own accord. Just because one stock becomes bad doesn't mean another stock is good. Also, there are no funny patterns in how well a stock is performing (how much it is paying in dividends). The stocks are not tricking you.

***Screen 15***

In the real experiment, we are only going to give you 1.5 seconds for each response (1.5 seconds to choose an Advisor, and 1.5 seconds to reveal how well a stock is performing). At the beginning that won't seem like very much time, and you may find the task difficult. Over time, as you learn to play, you will find that 1.5 seconds is enough time to make good decisions.

Our advice is to think carefully about your strategy, but also to trust your instincts. By concentrating, you can increase the number of points you win by a lot. There is an element of chance, but a lot of room for skill as well.

***Screen 16***

The Advisors will always stay on screen for 1.5 seconds, even after you already made a decision. Because of this, you will not be able to finish the experiment faster by making your decisions more quickly.

Every MTurk worker will complete the HIT in exactly the same time, so use the time you have for each decision to your advantage and earn as many points as possible!

***Screen 17***

You will get a bonus of approximately 1 cent for every 36 points you earn. The points can pile up quickly, and so will your bonus! Just do your best to earn as many points as possible!

The game lasts about 150 rounds and you will have 1.5 seconds for each choice. The entire study will take approximately 30 minutes.

You will be given a couple opportunities for breaks in the middle of the HIT.

The HIT will start after you press 'NEXT', so make sure you have your fingers on the 'F' and the 'J' keys, to choose one of the two Advisors. Remember to also press the SPACEBAR to reveal how well a stock is performing. Good luck!

**References**

Doll, B. B., Duncan, K. D., Simon, D. A., Shohamy, D., & Daw, N. D. (2015). Model-based

choices involve prospective neural activity. *Nature Neuroscience*, *18*, 767-772.

Gershman, S. J. (2016). Empirical priors for reinforcement learning models. *Journal of*

*Mathematical Psychology*, *71*, 1-6.

Kool, W., Gershman, S. J., & Cushman, F. A. (2017). Cost-benefit arbitration between multiple

reinforcement-learning systems. *Psychological Science*, *28*, 1321-1333.

Palminteri, S., Wyart, V., & Koechlin, E. (2017). The importance of falsification in

computational cognitive modeling. *Trends in cognitive sciences*, *21*, 425-433.

Shahar, N., Moran, R., Hauser, T. U., Kievit, R. A., McNamee, D., Moutoussis, M., ... & NSPN

Consortium. (2019). Credit assignment to state-independent task representations and its relationship with model-based decision making. *Proceedings of the National Academy of Sciences*, *116*, 15871-15876.

Stephan, K. E., Penny, W. D., Daunizeau, J., Moran, R. J., & Friston, K. J. (2009). Bayesian

model selection for group studies. *Neuroimage*, *46*, 1004-1017.

**Supplementary Tables**

Table S1.

*Parameter fits for the computational model.*

| **Statistic** | ***β*** | **α** | **λ** | ***w*** | ***π*** | ***ρ*** |
| --- | --- | --- | --- | --- | --- | --- |
| 25^th^ percentile | .58 | .80 | 0 | .70 | -.08 | -.40 |
| Median | .70 | 1 | .51 | .92 | .27 | -.07 |
| 75^th^ percentile | .90 | 1 | .75 | 1 | .86 | .19 |

Table S2.

*Coefficients for regression analysis of learning phase choice.*

| **Effect** | **Coefficient** | ***SE*** | ***z*** | ***p*** |
| --- | --- | --- | --- | --- |
| Intercept | 1.70 | 0.09 | 17.98 | < 2.00 × 10^-16^ |
| Reward | 1.47 | 0.07 | 19.80 | < 2.00 × 10^-16^ |
| Same State | 0.20 | 0.04 | 4.81 | 1.49 × 10^-06^ |
| Reward x Same State | 0.22 | 0.03 | 6.45 | 1.14 × 10^-10^ |

Table S3.

*Coefficients for regression analysis of post-learning evaluations. The weighting parameter was z-scored across participants prior to analysis; main effects of model-free values (QMF) and model-based values (QMB) can therefore be interpreted at the mean level of the weighting parameter (w = .83).*

| **Effect** | **Coefficient** | ***SE*** | ***df*** | ***t*** | ***p*** |
| --- | --- | --- | --- | --- | --- |
| Intercept | 4.73 | 0.11 | 63.39 | 44.64 | < 2.00 × 10^-16^ |
| QMF | 0.16 | 0.09 | 162.97 | 1.82 | 0.07 |
| QMB | 0.30 | 0.14 | 71.46 | 2.17 | 0.03 |
| W | 0.07 | 0.11 | 64.45 | 0.63 | 0.53 |
| QMF x W | -0.24 | 0.08 | 148.01 | -2.97 | 0.004 |
| QMB x W | -0.12 | 0.14 | 67.78 | -0.90 | 0.37 |
